# Non-invasive endometrial assessment: shear wave elastography in diagnosing endometrial hyperplasia

**DOI:** 10.1590/1806-9282.20250237

**Published:** 2025-10-27

**Authors:** Uğurcan Zorlu, Sezer Nil Yılmazer Zorlu, Burak Elmas

**Affiliations:** 1 Ankara Bilkent City Hospital, Department of Obstetrics and Gynecology – Ankara, Turkey.; 2 Ankara Mamak State Hospital, Department of Radiology – Ankara, Turkey.

**Keywords:** Diagnosis, Elasticity imaging techniques, Endometrial hyperplasia, Metrorrhagia, Ultrasonography

## Abstract

**OBJECTIVE:**

Endometrial hyperplasia is a precursor lesion that may progress to endometrial carcinoma, particularly in cases with atypia. Early and accurate diagnosis is essential for timely intervention. The aim of the study was to evaluate the diagnostic performance of shear wave elastography in differentiating normal endometrium, non-atypical hyperplasia, and atypical hyperplasia in premenopausal women with abnormal uterine bleeding.

**METHODS:**

This prospective study included 235 premenopausal women over 45 years of age with abnormal uterine bleeding. All patients underwent transvaginal ultrasonography and shear wave elastography using a Toshiba Aplio 500 system. Five regions of interest were placed within heterogeneous endometrial areas, and mean shear wave elastography (mean-SWE) and highest shear wave elastography (highest-SWE) values were recorded. Histopathological evaluation was performed via probe curettage, and patients were categorized into three groups: normal endometrium, non-atypical hyperplasia, and atypical hyperplasia. Receiver operating characteristic curve analysis and logistic regression were conducted.

**RESULTS:**

Mean-SWE and highest-SWE values were significantly higher in the hyperplasia groups compared to the normal endometrium group (p=0.021, p=0.001). Highest-SWE had the highest diagnostic accuracy (cut-off: 37.71 kPa, sensitivity: 85.9%, specificity: 48.0%). Logistic regression identified body mass index, uterine free fluid, mean-SWE, and highest-SWE as independent predictors of endometrial hyperplasia (p<0.05).

**CONCLUSIONS:**

Shear wave elastography demonstrated significant potential as a non-invasive tool for diagnosing endometrial hyperplasia. Mean-SWE and highest-SWE were independent predictors, supporting shear wave elastography as a complementary method to transvaginal ultrasonography. Future research should optimize shear wave elastography protocols for gynecological assessments.

## INTRODUCTION

Endometrial hyperplasia is a pathological condition characterized by abnormal proliferation of the endometrial glands, often presenting as abnormal uterine bleeding (AUB) in premenopausal and postmenopausal women. If left untreated, endometrial hyperplasia, particularly with atypia, may progress to endometrial carcinoma, making early detection and accurate diagnosis crucial^1^. Traditionally, endometrial assessment relies on transvaginal ultrasonography (TVUS) and endometrial biopsy. However, biopsy is an invasive procedure, and TVUS has limitations in distinguishing between benign and malignant endometrial pathologies^2^.

In recent years, shear wave elastography (SWE) has emerged as a promising non-invasive imaging technique that quantifies tissue stiffness by measuring the propagation speed of shear waves through the tissue. Stiffness alterations in the endometrium may reflect underlying histopathological changes, providing additional diagnostic value beyond conventional ultrasonography^3^. Studies suggest that SWE can differentiate between normal, hyperplastic, and malignant endometrial tissues by assessing elasticity parameters such as mean-SWE and highest-SWE^4^.

Several prospective studies have evaluated the efficacy of SWE in diagnosing endometrial lesions. A recent meta-analysis highlighted that SWE significantly improves the sensitivity and specificity of diagnosing endometrial hyperplasia and cancer when combined with TVUS^5^. Moreover, the integration of elastographic techniques into routine gynecological evaluations may reduce the need for unnecessary biopsies and optimize patient management strategies.

This study aims to assess the diagnostic value of SWE in evaluating endometrial hyperplasia in premenopausal women with AUB. By comparing SWE findings with histopathological results obtained through probe curettage, we aim to determine the reliability of SWE in distinguishing between different endometrial pathologies. We hypothesize that SWE, as a quantitative and non-invasive tool, may enhance the diagnostic accuracy for endometrial hyperplasia and provide a valuable alternative to traditional methods.

## METHODS

This prospective study was conducted in the Gynecology Department of Ankara City Hospital in 2024 and included premenopausal women over the age of 45 who presented with AUB. The study was initiated after obtaining informed consent from all patients and receiving approval from the Ethics Committee of Ankara Bilkent City Hospital (Ethics Committee No. TABED 2-24-668). The study population was carefully selected to ensure homogeneity, and patients with a history of hormonal therapy, oral contraceptive use, or prior medical treatment for endometrial pathology were excluded. Additionally, individuals with coagulopathies, endometrial polyps, adenomyosis, uterine fibroids, thyroid dysfunctions, or other hormonal disorders were not included. Patients with ultrasonographically suspected or hysteroscopically visualized endometrial polyps were excluded from the study. This decision was based on the biomechanical heterogeneity of polyps, which exhibit distinct fibroglandular elasticity characteristics that could confound SWE measurements, particularly when using multiple regions of interest (ROIs). All participants underwent a detailed clinical evaluation, including body mass index (BMI) calculation, measurement of cancer antigen (CA)-125 levels, and assessment of associated conditions such as diabetes and polycystic ovary syndrome (PCOS). Smoking status was also recorded.

TVUS was performed on all patients to assess endometrial thickness and evaluate structural abnormalities. In addition to TVUS, SWE was employed as a non-invasive imaging method to quantitatively assess endometrial stiffness. Endometrial thickness (ET) was measured using TVUS during the same session as SWE, specifically during the mid-cycle phase to ensure consistency across patients. All SWE examinations were performed using a Toshiba Aplio 500 ultrasound system. Five ROIs were manually placed within the heterogeneous areas of the endometrium, ensuring an accurate representation of tissue stiffness. The system automatically calculated the maximum SWE value (highest SWE), while the mean SWE value was determined based on the five ROIs. All measurements were expressed in kilopascals (kPa), with the device's quality factor (reliability measure index, RMI) maintained within an optimal range of 0.4–1.0 kPa to ensure reliable data acquisition.

Following sonographic and elastographic evaluation, all patients underwent endometrial sampling via probe curettage for histopathological examination. Endometrial tissue sampling was performed using a Novak endometrial curette under transabdominal ultrasonographic guidance. The procedure was conducted by an experienced gynecologist to ensure the accuracy and representativeness of the sample. Real-time ultrasound allowed for optimal positioning of the curette within the uterine cavity and enhanced the likelihood that the sampled area corresponded to the region assessed via SWE. This technique, widely accepted in clinical gynecology, provided sufficient tissue for histopathological analysis and minimized sampling bias. The approach aligns with standard diagnostic protocols for evaluating AUB in perimenopausal women. Although hysteroscopic biopsy is recognized as the gold standard for localized lesions, due to the real-world constraints of resource availability and clinical throughput in a public hospital setting, we adopted a more accessible and cost-effective method. The technique applied is consistent with common outpatient gynecological practice and is supported by literature demonstrating high diagnostic concordance between blind curettage and hysteroscopy in diffuse endometrial pathology^6^. Based on pathology results, participants were categorized into three groups: those with normal endometrium, those diagnosed with non-atypical endometrial hyperplasia, and those with atypical endometrial hyperplasia. Statistical analyses were conducted using SPSS (Statistical Package for the Social Sciences), version 25.0. The normality of continuous variables was assessed using the Shapiro-Wilk test, which confirmed a normal distribution, allowing for the use of parametric statistical tests. Comparisons between the three groups were performed using one-way analysis of variance for continuous variables, while categorical variables were analyzed with the chi-square test.

To evaluate the diagnostic performance of SWE in predicting endometrial hyperplasia, receiver operating characteristic (ROC) curve analysis was conducted. The area under the curve (AUC), optimal cut-off values, sensitivity, specificity, positive predictive value (PPV), and negative predictive value (NPV) were calculated for both mean SWE and highest SWE parameters. Additionally, multivariate logistic regression analysis was performed to identify independent predictors of endometrial hyperplasia. Odds ratios (OR) and 95%CI were reported, with a significance level set at p<0.05.

## RESULTS

The study included 235 premenopausal women over the age of 45 who presented with AUB. Based on histopathological examination, 171 patients (72.7%) had normal endometrium, 41 patients (17.4%) had non-atypical endometrial hyperplasia, and 23 patients (9.8%) were diagnosed with atypical endometrial hyperplasia.

The mean age of patients was comparable across the three groups and did not show a statistically significant difference (*p*=0.124). Similarly, gravida and parity values were not significantly different among the groups (*p*=0.233 and *p*=0.254, respectively). However, BMI was significantly higher in the hyperplasia groups compared to the normal endometrium group (*p*=0.041). Smoking prevalence was higher in patients with non-atypical hyperplasia (34.1%) compared to the normal endometrium group (33.3%) and atypical hyperplasia group (30.4%), with a statistically significant difference (*p*=0.039). Similarly, CA-125 levels were significantly elevated in patients with atypical hyperplasia (37.6±29.3 U/mL) compared to the other groups (*p*=0.020).

Although the mid-cycle endometrial thickness appeared higher in the hyperplasia groups, this difference did not reach statistical significance (p=0.071). This finding highlights the limited diagnostic performance of endometrial thickness alone and emphasizes the potential added value of SWE in differentiating endometrial pathologies when conventional ultrasonographic parameters are inconclusive.

The presence of diabetes and uterine free fluid was more common in patients with hyperplasia, with a significant difference among the groups (*p*=0.024 and *p*=0.019, respectively). PCOS prevalence was highest in the atypical hyperplasia group (17.3%), showing a statistically significant association (*p*=0.008). Mean SWE and Highest SWE values were significantly different across the three groups. Patients with hyperplasia had higher mean SWE and highest SWE values than those with normal endometrium (*p*=0.021 and *p*=0.001, respectively). The demographic and clinical characteristics of the study groups are presented in Table 1.

**Table 1:** Demographic and clinical characteristics of the study groups.

Parameter	Normal endometrium (n=171)	Non-atypical endometrial hyperplasia (n=41)	Atypical endometrial hyperplasia (n=23)	*p*
Age (years)	48.13±2.66	47.54±3.01	47.52±3.42	0.124
Gravida	2.67±1.1	2.54±0.9	2.51±0.9	0.233
Parity	2.11±0.9	2.13±1	2.04±0.9	0.254
BMI (kg/m^2^)	26.41±5.29	27.12±5.66	27.15±6.01	0.041
Mid-cycle endometrial thickness (mm)	15.6±3.22	16.01±3.51	17.04±3.66	0.071
Smoking (%)	33.3	34.1	30.4	0.039
CA-125 (U/mL)	33.9±26.7	32.6±30.9	37.6±29.3	0.020
Diabetes (%)	10.6	12.19	17.3	0.024
Uterine free fluid (%)	11.3	12.19	17.3	0.019
PCOS (%)	7.8	14.6	17.3	0.008
Mean SWE (kPa)	16.04±8.99	24.52±11.49	27.88±15.42	0.021
Highest SWE (kPa)	31.00±15.41	39.70±13.20	42.30±15.79	0.001

BMI: body mass index; PCOS: polycystic ovary syndrome; SWE: shear wave elastography.

ROC curve analysis was performed to evaluate the diagnostic accuracy of mean SWE and Highest SWE in ­differentiating normal endometrium from hyperplasia and distinguishing non-atypical hyperplasia from atypical hyperplasia.

For differentiating normal endometrium from hyperplasia, the mean SWE cut-off was 24.33 kPa, with a sensitivity of 48.4% and specificity of 84.2%. The highest SWE cut-off was 37.71 kPa, yielding 85.9% sensitivity and 48.0% ­specificity. For differentiating non-atypical hyperplasia from atypical hyperplasia, the mean SWE cut-off was 26.40 kPa, with 87.0% sensitivity and 49.5% specificity. The highest SWE cut-off was 41.59 kPa, achieving 78.3% sensitivity and 63.4% ­specificity. The results of the ROC analysis are presented in Table 2.

**Table 2:** Receiver operating characteristic analysis results for shear wave elastography parameters.

Parameter	AUC	Cut-off (kPa)	Sensitivity (%)	Specificity (%)	PPV (%)	NPV (%)
Mean SWE (normal vs. hyperplasia)	0.651	24.33	48.4	84.2	74.0	52.0
Highest SWE (normal vs. hyperplasia)	0.716	37.71	85.9	48.0	62.0	77.0
Mean SWE (non-atypical vs. atypical hyperplasia)	0.574	26.40	87.0	49.5	63.0	79.0
Highest SWE (non-atypical vs. atypical hyperplasia)	0.698	41.59	78.3	63.4	68.0	74.0

AUC: area under the curve; PPV: positive predictive value; NPV: negative predictive value; SWE: shear wave elastography.

Multivariate logistic regression analysis identified uterine free fluid presence, BMI, mean SWE, and highest SWE as independent predictors of endometrial hyperplasia. Higher BMI values were associated with increased odds of hyperplasia (OR 1.075, *p*=0.025). Mean SWE (OR 1.065, *p*=0.001) and highest SWE (OR 1.051, *p*=0.001) were significant predictors of hyperplasia. However, CA-125 levels, PCOS, diabetes, and smoking were not statistically significant predictors. These results are summarized in Table 3.

**Table 3:** Logistic regression analysis for predicting endometrial hyperplasia.

Model	OR	95%CI (lower)	95%CI (upper)	*p*
PCOS presence	1.766	0.602	5.183	0.301
Diabetes presence	1.464	0.508	4.222	0.481
Uterine free fluid	2.575	1.069	6.201	0.035
CA-125	1.002	0.985	1.009	0.634
Smoking	0.651	0.308	1.377	0.261
BMI	1.075	1.009	1.145	0.025
Mean SWE	1.065	1.029	1.103	0.001
Highest SWE	1.051	1.027	1.075	0.001

OR: odds ratio; CI: confidence interval; PCOS: polycystic ovary syndrome; BMI: body mass index; SWE: shear wave elastography.

## DISCUSSION

This study investigated the role of SWE in diagnosing endometrial hyperplasia in premenopausal women presenting with AUB. The findings suggest that SWE parameters, particularly mean SWE and highest SWE, are valuable in differentiating normal endometrium from hyperplasia and further distinguishing non-atypical hyperplasia from atypical hyperplasia. These results align with recent studies indicating that tissue stiffness measurements obtained through elastography can enhance the diagnostic accuracy of endometrial abnormalities^1,4^.

One of the key findings of this study was that patients with endometrial hyperplasia exhibited significantly higher mean SWE and the highest SWE values compared to those with normal endometrium. This suggests that increased endometrial stiffness may serve as an important biomarker for hyperplasia, which is consistent with previous reports emphasizing the potential of SWE in gynecological imaging^2,5^. Moreover, the differences in elastographic parameters between non-atypical and atypical hyperplasia highlight the utility of SWE in risk stratification, potentially aiding in the early identification of patients who may require closer monitoring or intervention.

The ROC analysis further supports the diagnostic value of SWE, demonstrating that both mean SWE and highest SWE yielded moderate-to-high accuracy in distinguishing normal endometrium from hyperplasia and in differentiating between non-atypical and atypical hyperplasia. The observed sensitivity and specificity values suggest that the highest SWE, in particular, may have a stronger predictive ability, as also indicated in previous studies^7,8^. These findings underscore the role of SWE as a non-invasive, reproducible imaging technique that can complement conventional ultrasonography in evaluating endometrial pathology.

In addition to SWE parameters, multivariate logistic regression analysis identified BMI, uterine free fluid, and SWE values as independent predictors of endometrial hyperplasia. The association between higher BMI and increased risk of endometrial hyperplasia aligns with established knowledge, as obesity is recognized as a key risk factor for endometrial pathology due to unopposed estrogen exposure^9^. Similarly, the presence of uterine free fluid as a significant predictor suggests that it may reflect underlying pathological changes within the endometrium. The strong association of mean SWE and highest SWE with hyperplasia further supports the notion that stiffness alterations in the endometrium may provide critical diagnostic insights.

The exclusion of patients with endometrial polyps was methodologically deliberate. While polyps are indeed prevalent in perimenopausal women and may coexist with or mimic hyperplastic changes, their localized and structurally distinct nature introduces considerable variance in SWE stiffness values. Moreover, as our study was designed to differentiate among histologically diffuse categories of endometrial pathology (normal endometrium, non-atypical hyperplasia, and atypical hyperplasia), the inclusion of focal lesions such as polyps could have impaired the interpretability and statistical power of our comparative analysis. Nevertheless, we acknowledge that a separate investigation focusing on the elastographic features of endometrial polyps would be of clinical relevance and recommend this as a future research direction.

While the results of this study demonstrate the potential of SWE in enhancing the non-invasive assessment of endometrial hyperplasia, certain limitations should be acknowledged. First, the study cohort consisted only of premenopausal women over 45 years of age, which may limit the generalizability of the findings to younger age groups or postmenopausal patients. Additionally, although SWE has demonstrated promising results, variations in measurement techniques and operator dependency remain potential challenges in standardizing its clinical application^10^. Further multicentric studies with larger sample sizes and longitudinal follow-ups are needed to validate these ­findings and explore the prognostic implications of elastographic parameters in endometrial pathology.

One of the limitations of this study is the absence of a hysteroscopically guided biopsy. This may have reduced diagnostic sensitivity, particularly in detecting focal atypical lesions. However, recent studies have demonstrated significant diagnostic concordance between blind dilatation and curettage (D&C) and hysteroscopic biopsy in the presence of diffuse endometrial pathology. In a study by Narayan et al., a high level of correlation was reported between the pathological diagnoses obtained via blind D&C and those acquired through hysteroscopy in patients evaluated for AUB^6^.

These findings suggest that while hysteroscopy may indeed improve diagnostic accuracy in the targeting of focal lesions, carefully performed blind curettage remains a valid, cost-effective, and accessible method in many clinical settings. Nonetheless, we fully acknowledge the limitations associated with this technique. Therefore, we propose that future studies integrating SWE with hysteroscopically targeted biopsy could further enhance diagnostic precision.

## CONCLUSION

This study highlights the diagnostic utility of SWE in ­differentiating normal endometrium from hyperplasia and in identifying atypical hyperplasia. The ability of SWE parameters to serve as independent predictors of hyperplasia suggests that SWE may be a valuable adjunct to traditional imaging modalities in the evaluation of endometrial abnormalities. Future research should focus on optimizing SWE protocols and integrating elastography into standardized clinical workflows for early and accurate detection of endometrial pathology.

## Data Availability

Patient data is stored indefinitely in the hospital's automation system. It can be shared upon request, provided that patient's identity remains confidential. The datasets generated and/or analyzed during the current study are available from the corresponding author upon reasonable request.
